# Quantitative Evaluation of Psychological Tolerance under the Haze: A Case Study of Typical Provinces and Cities in China with Severe Haze

**DOI:** 10.3390/ijerph19116574

**Published:** 2022-05-27

**Authors:** Haiyue Lu, Xiaoping Rui, Gadisa Fayera Gemechu, Runkui Li

**Affiliations:** 1 College of Hydrology and Water Resources, Hohai University, Nanjing 210003, China; 191309010002@hhu.edu.cn (H.L.); gadisa@hhu.edu.cn (G.F.G.); 2 College of Earth and Engineering, Hohai University, Nanjing 211100, China; 3 Faculty of Natural Sciences, Salale University, Fiche 245, Ethiopia; 4 College of Resources and Environment, University of Chinese Academy of Sciences, Beijing 100049, China

**Keywords:** sentiment analysis, haze perception, quantitative evaluation, psychological tolerance level, spatio-temporal trajectory

## Abstract

The interplay of specific weather conditions and human activity results due to haze. When the haze arrives, individuals will use microblogs to communicate their concerns and feelings. It will be easier for municipal administrators to alter public communication and resource allocation under the haze if we can master the emotions of netizens. Psychological tolerance is the ability to cope with and adjust to psychological stress and unpleasant emotions brought on by adversity, and it can guide human conduct to some extent. Although haze has a significant impact on human health, environment, transportation, and other factors, its impact on human mental health is concealed, indirect, and frequently underestimated. In this study, psychological tolerance was developed as a psychological impact evaluation index to quantify the impact of haze on human mental health. To begin, data from microblogs in China’s significantly haze-affected districts were collected from 2013 to 2019. The emotion score was then calculated using SnowNLP, and the subject index was calculated using the co-word network approach, both of which were used as social media evaluation indicators. Finally, utilizing ecological and socioeconomic factors, psychological tolerance was assessed at the provincial and prefecture level. The findings suggest that psychological tolerance differs greatly between areas. Psychological tolerance has a spatio-temporal trajectory in the timeseries as well. The findings offer a fresh viewpoint on haze’s mental effects.

## 1. Introduction

Although haze pollution in China has decreased in recent years, it still occurs frequently in northern areas with unique industrial structures, causing considerable disruption to people’s lives. Water, dust, water vapor, and smoke in the air cause haze, which reduces horizontal visibility to less than 10 km [1,2]. The fundamental cause of haze is a particulate matter with a dynamic equivalent diameter of less than or equal to 2.5 microns [3]. According to studies, over 1.6 billion people worldwide are affected by urban air pollution [4]. The number of cardiovascular, pulmonary, dermatological, and other hospital departments has increased because of smog [5]. People who spend a long period in such an atmosphere face serious health concerns, which can lead to a variety of disorders [6]. Furthermore, air pollution might endanger human mental health in both the natural environment and the indoor environment [7]. When the weather is terrible, people are more likely to be pessimistic, which can lead to sadness, an increased risk of negative conduct, and a decrease in subjective well-being [8,9]. Weibo is popular as a “cloud communication” tool because of its short content and real-time communication. Weibo is a data source with high research value since it can generate a large number of multi-source and real-time information in a short amount of time [10]. Tao [9] analyzed the content and sentiment of Weibo comments about the city’s air quality and tourists’ perceptions of air quality to contribute to the city’s building. Jia [11] used the simultaneous equation model to examine microblog data and the impact of social media on environmental governance. Wang et al. [12] used microblog check-in data to investigate the temporal and spatial aspects of tourism flow in Lanzhou, which is beneficial to the rational development of tourism resources. A huge number of haze-related theme microblogs will be generated in the case of haze. These wide data can be utilized to better understand people’s perceptions of haze and the effects of haze on mental health. The ability to tolerate and adjust to psychological strain and bad emotions brought on by adversity is referred to as psychological tolerance. When haze occurs, people’s psychological tolerance will vary depending on pollution levels and individual sensitivity. Varied Weibo numbers, themes, and sentiment trends reveal that people have different psychological proclivities when it comes to social media.

This study employs relevant Weibo data and unearths useful information to provide a reference for resource allocation and urban management in the event of haze. It also takes into account the topics of concern, the number of Weibo users and their emotions, and employs multiple Weibo datasets to assess the psychological impact of haze to provide an effective reference for the establishment of psychological early warning mechanisms in the event of haze.

### 1.1. Sentiment Analysis

Sentiment analysis, also known as opinion mining and tendency analysis, uses data mining and natural language processing (NLP) technologies to analyze, process, judge, and summarize subjective texts with emotional color [13]. It can be used to understand people’s attitudes toward a social phenomenon or their preferences for something [14], which is very important for opinion research, information prediction, and artificial intelligence. There are two kinds of sentiment analysis for microblog data: machine-learning algorithms and sentiment lexicon. It is very important in the fields of opinion judgment, information prediction, and artificial intelligence [15,16].

To calculate scores and determine emotional polarity, the sentiment lexicon approach uses an emotion dictionary [17]. Shashank et al. [18] suggested “SentiDraw”, a new approach based on traditional Pointwise Mutual Information (PMI) and star rating. In terms of emotion classification, this method is more accurate. By computing semantic similarity, Araque et al. [19] built a sentiment classification model with good classification performance. Abdulmohsen et al. [20] developed a Saudi dialect emotion dictionary (SauDiSenti) based on a traditional emotion dictionary that had a positive impact on emotion analysis. The naive Bayes classifier, support vector machine, maximum entropy, and other machine learning classifiers are currently in use [21]. Alsayat [22] employed word-embedding technology to develop the LSTM network, which has high classification accuracy and can understand new, unusual words. Uni et al. [23] used an aspect-based emotion analysis model to achieve automatic emotion categorization, which is superior to typical deep learning architecture. Garcia-ordas et al. [24] suggested an emotion analysis algorithm that can handle any length of audio to realize audio data emotion analysis in real time.

### 1.2. Mental Health Impact Assessment

Haze weather was featured in the official natural catastrophes list in 2014, and it turned out to be terrible weather. As a result, haze has long been a hotbed for academic research. In terms of the impact of haze on mental health, Song et al. [25] used the probit model to examine the impact of haze on human subjective happiness and discovered that inhabitants in heavy haze locations with higher income levels were happier. Using two rounds of cross-sectional survey data from 92 Chinese cities, Wang et al. [26] investigated the impact of haze pollution and commuting behavior on life satisfaction. Li et al. [27] conducted a questionnaire study on 605 pregnant women in Nanjing to assess the influence of PM2.5 exposure and risk perception on the psychological stress of pregnant women, and corresponding recommendations were made, such as encouraging pregnant women to exercise outside in sunny weather.

Previous research has largely focused on emotional attitudes during a certain period or during a hot event and has rarely looked at changes in emotional attitudes over time or the spatial disparities between different regions. When it came to studies on the psychological effects of haze, the majority of them used a simple sampling survey to do a rough emotional analysis rather than conduct an in-depth quantitative characterization. This study is based on the analysis of subjective text, lengthy timeseries, and spatial heterogeneity elements as well as the long-term trend of public opinion events. The goal of this work was to perform a spatio-temporal analysis of haze emotions using a simple Bayesian-based emotion mining algorithm, investigate ways of quantitatively characterizing psychological effects, and quantify the psychological impact index of the haze.

## 2. Materials and Methods

### 2.1. Study Area

**Figure 1:**
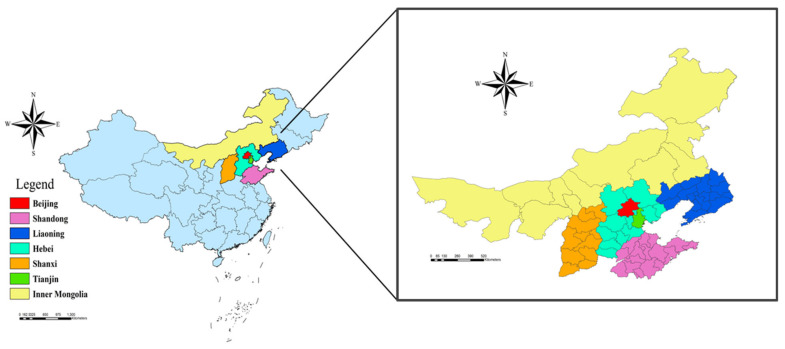
The geographical location of the study area.

Beijing, Tianjin, Shanxi, Hebei, Inner Mongolia, Liaoning, and Shandong provinces are included in the study area because they are significantly more seriously contaminated by haze and are typical research sites for “haze”-related research. The Beijing-Tianjin-Hebei region is part of the “Capital Economic Circle”, which governs Beijing, Tianjin, and several cities in Hebei Province. Because of its unique physical environment and development conditions, this region was once known as China’s foggiest, so choosing it as the research area is significant. At the same time, Shanxi Province, Shandong Province, Inner Mongolia Province, and Liaoning Province are chosen as the references, which have comparative relevance, using this region as the center and expanding outwards. Figure 1 depicts the study area’s geographical position.

### 2.2. Datasets and Data Processing

#### 2.2.1. Data Collection

This research primarily uses two types of data: air quality data and data from microblogs. The former is derived from the air quality index website (https://aqicn.org/map/china/cn/) (accessed on 16 April 2020), which was used to collect data from 2013 to 2019. Because this season’s fog is more severe than prior seasonal fogs, the related Weibo data are richer, more representative, and of greater study value. City, date, PM2.5, PM10, carbon monoxide, nitrogen dioxide, and sulfur dioxide levels are all included in the data. This study uses PM2.5 content to depict the degree of haze pollution because PM2.5 is the main cause of haze pollution. PM2.5 data have a one-hour temporal resolution and a city-scale geographical resolution. The unit used in this study is µg/m^3^, and the PM2.5 concentration is averaged to get the mean value of PM2.5 in winter every year. The content of each microblog is captured by a descendent collector [28], which includes the user ID, microblog, time range, amount of likes and comments, and so on. Weibo data have a minute temporal resolution and a city-sized geographical resolution.

#### 2.2.2. Sample Data Collection and Processing

Microblog data are unstructured data with a great deal of noise, such as repeated data, commercial adverts, special symbols, and so on, which a computer cannot handle directly. Preprocessing processes such as data cleaning, text segmentation, and stop word filtering can increase accuracy and efficiency. In this paper, Python was used to remove emojis, labels, and weblinks, while Jieba word segmentation tool was used to partition data into words. The hit-stop words table is then loaded to filter “me, you, he”, and other stop words before the original microblog data are turned into structured data made up of many words. Table 1 shows the outcomes of the data preprocessing.

#### 2.2.3. Other Data

Indicators of the ecological environment and social economy, mostly from the urban statistics yearbook, are among the other data sources. Indicators were collected from 2013 to 2019 (winter). Ecological environmental indicators include air quality (µg/m^3^), temperature (°C), relative humidity (percent), wind speed (m/s), and pollution days (d), with a month as the temporal resolution and a province as the spatial resolution. Population density (person/km^2^), per capita GDP (yuan), the proportion of secondary industry (percent), the proportion of built-up area (percent), road area (10,000 m^2^), per capita green space area (m^2^), education level, and gender ratio (male = 100) are some of the social economic indicators. The temporal resolution of these indicators is a month, and the spatial resolution is a province.

### 2.3. Research Method

**Figure 2:**
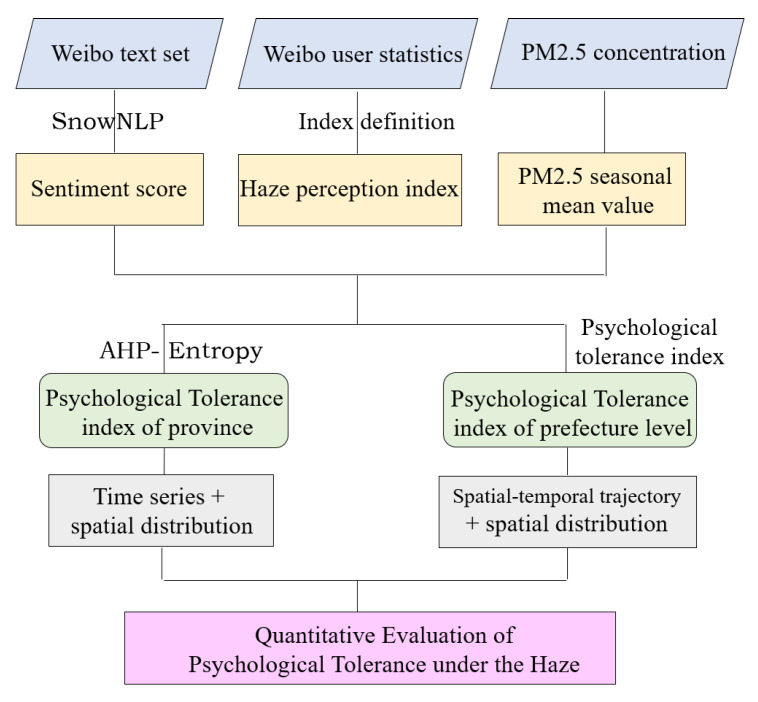
A flowchart depicting the techniques used.

This study aims at the quantitative evaluation of psychological tolerance in the case of haze. Firstly, the SnowNLP model is used to calculate the sentiment score, the number of microblog users, and the number of microblogs, and other indicators are used to define the haze perception index and calculate PM2.5 seasonal mean value. Based on this, we integrated other index data to realize the evaluation of psychological tolerance at provincial scale and prefecture level city scale, respectively, and clarify its temporal and spatial characteristics. The method flowchart is shown in Figure 2.

#### 2.3.1. SnowNLP

The naive Bayes algorithm [29] is based on the Bayes theorem and assumes that each condition feature is independent of the others [30]. SnowNLP is a Chinese natural language-processing package that includes features such as Chinese word segmentation, emotion analysis, text keyword extraction, post-tagging, text classification, and text abstract extraction [31]. In terms of emotion analysis, the SnowNLP library features a built-in emotion analysis model that was trained in advance using commodity review data, but it does not work well with microblog data. As a result, this study manually highlighted the emotional tendencies of 28,000 microblog texts, and the emotion analysis model was retrained to fit the needs of this study.

To streamline the corpus and calculate each word occurrence probability, P (word), and the probability of different categories of words appearing, P (word|category), we used Python programming language to read microblog Excel data, called the pre-trained SnowNLP model; training data calculation based on the labeled with complete prior probability, P (category); and, finally, calculated target words belonging to the positive or negative posterior probability, P (category|word), to determine the ultimate emotion rating is determined by the estimated likelihood value. The result of the SnowNLP emotion analysis is a number between 0 and 1. When the result exceeds 0.5, it means that emotion is skewed toward pleasant feelings. The more positive the emotion, the higher the value. When the value is less than 0.5, on the other hand, the emotion is more likely to be negative. The more negative the emotion, the lower the value [32].

#### 2.3.2. AHP-Entropy Method

The analytic hierarchy process (AHP) was proposed by American operational research scientist Sass in the 1970s [33]. It is an evaluation process that combines qualitative and quantitative methods. The specific analytic procedure [34] is as follows: problems are decomposed in top-down order into target-rules-scheme layers, and the degree of correlation and relative relevance of components at each layer can be determined in some way. The basic idea is to divide complex problems into hierarchical structures, use the scale of proportion to compare the relative importance of factors at two levels, create a comparative judgment matrix for two levels, solve the corresponding weight, and choose the weight calculation method to calculate the total weight of the overall target layer [35,36,37]. The following processes are involved in using AHP to analyze complicated problems: creating a hierarchical structure model, creating a factor attribute judgment decision table, solving judgment matrix weight, and calculating and sorting comprehensive weight.

The primary premise of the entropy method is to divide the overall assessment target layer into the index of the lower level and then to evaluate the overall objective by evaluating a single factor. The AHP method is logical, but the process of judging the relative importance degree among the index factors when constructing the judgment matrix is subjective. As a result, the entropy weight method was introduced in this study, which uses the entropy magnitude of the index to determine the relative weight, reducing the subjectivity of AHP and improving the assessment model’s reliability. Entropy was first created from the notion of thermodynamics in physics, and it is now widely explored in numerous domains [38,39,40]. It is mostly used to reflect the degree of chaotic measure of a given system. Entropy is a measure of how much information is there, and the more information a given indicator component holds, the more relevant it is for the evaluation. When the gap between the assessment objectives on a particular indicator is considerable, the smaller the entropy, the more information the indicator factor has, and the greater its weight in the evaluation system.

(1) Matrix of Index Judgment

Experts assessed the relative value of indicators to create a judgment matrix. The feature vector corresponding to its greatest feature value is calculated on this basis, and the component vector of this vector relates to the weight of indicators and the judgment matrix’s assessment criteria, as shown in Table 2.

The upper (lower) half matrix and the lower (upper) half matrix of the matrix are reciprocal, and the diagonal number is 1. If the element of the *i*-row and *j*-column of the upper half matrix is *a_ij_*, the off-duty matrix corresponds to an element where *i* represents the index’s row number, and *j* represents the index’s column number.

(2) Calculation of Feature Vectors and a Consistency Test

This technique is highly subjective, as the aforementioned index judgment matrix provides subjective scores based on expert opinions, and as a result, the index of consistency of *CR* value is used to quantitatively test the matrix’s scientific rationality, and the *CR* test’s main formula is provided in Equation (1):

(1) $$CR=\frac{CI}{RI}$$

(2) $$CI=\frac{\lambda_{max}}{n-1}$$

The numerator *CI* is used to test the matrix’s consistency, whereas *RI* is used to test the matrix’s average random consistency, which is a constant that relies on the order. Table 3 shows the most generally used order matching the *RI* value. In the *CI* test formula, *n* denotes the matrix order, and *max* is the typical root, which is computed from the matrix. When the *CR* value is less than 0.1, the matrix is regarded as consistent; when the *CR* value is greater than 0.1, the matrix is considered inconsistent, and the judgment matrix should be rejected or adjusted properly.

(3) Calculate the weight of the index entropy

The index with % as a unit is zeroed out throughout the standardization process, and the value of 0 after normalized standardization is substituted. Then, 0 is replaced with 0.01, and the difference can be ignored to make the calculation result as close to the objective fact as possible.

Calculate the weight *P_ij_* of the *i*th evaluation object under the *j*th index factor:

(3) $$P_{ij}=Y_{ij}/\overset{n}{\underset{i=1}{\sum }}Y_{ij}$$

where *P_ij_* is the weight of the *i*th evaluation object under the *j*th index factor.

Calculate the entropy of the index:

(4) $$e_{ij}=-\frac{1}{ln\;n}\overset{m}{\underset{i=1}{\sum }}P_{ij}lnP_{ij}$$

where $e_{ij}$ is the entropy value of the *j*th evaluation index factor, and *m* is the number of evaluation objects.

Finally, calculate the entropy weight of the index: $S_{j}=\frac{1-e_{j}}{\sum_{j=1}^{n}1-e_{j}}$, $S_{j}$ is the entropy weight of the *j*th evaluation index factor.

(4) Weight of the Combination

The comprehensive weight of the index is obtained by synthesizing the subjective weight and the objective weight, $\omega_{i}=\frac{{\left(\omega_{1i}\omega_{2i}\right)}^{0.5}}{\sum_{i=1}^{m}{\left(\omega_{1i}\omega_{2i}\right)}^{0.5}}$, where $\omega_{1i}$ is the subjective weight of the indicator, $\omega_{2i}$ is the objective weight of the indicator, $\omega_{i}$ is the comprehensive combination weight of the indicator, and *i* = 1, 2, 3,…, *m*. The Lagrangian operator is introduced in the calculation for optimization to ensure that the $\omega_{i}$ is close to the $\omega_{1i}$ and the $\omega_{2i}$.

#### 2.3.3. Haze Psychological Tolerance Index

A tolerance index was proposed in this study as the basis for a quantitative assessment of psychological tolerance at the prefecture level. To quantitatively assess people’s psychological tolerance under haze conditions and analyze the psychological impact of haze on individuals, the number of microblogs, emotional inclination, and air quality index were used as evaluation indices. The tolerance index is determined using the following formula:

(5) $$T=\frac{W_{E_{p}\times }A_{E_{p}}}{W_{E_{N}\times }\left(1-A_{E_{N}}\right)\times C_{PM_{2.5}}}$$

where T is the tolerance index, $W_{E_{p}}$ is the number of positive microblogs, $W_{E_{N}}$ is the number of negative microblogs, $A_{E_{p}}$ is the mean positive sentiment score, $A_{E_{N}}$ is the mean negative sentiment score, and $C_{PM_{2.5}}$ is the PM2.5 concentration. In a hazy condition, the larger the index, the higher people’s psychological tolerance is; otherwise, tolerance is poor. Meanwhile, the change trend and rule of psychological tolerance may be determined by comparing the tolerance index in different years. The number of microblogs is based on statistical analysis, the emotional value is computed using the SnowNLP model, and the PM2.5 concentration is based on the average processing of the original data. These variables are counted and calculated, and the psychological tolerance index is calculated using the algorithm above.

#### 2.3.4. Method of Spatiotemporal Trajectory

Initially, the spatial-temporal trajectory method was widely employed in land-use studies. It is a technique for analyzing spatio-temporal dynamic changes in a given attribute and estimating spatio-temporal properties of continuous or discontinuous occurrences [41]. The change of the study item (phenomenon or thing) has special time continuity in space according to this technique [42,43,44,45,46,47,48,49,50,51], and the spatio-temporal change track of the grid can be represented by changing numbers or characters, such as CCBC, 1232, 3212, and so on. Each code represents the research object’s change kind during the change procedure. The combination of codes can be used to obtain not only the state information of the study item in a given time sequence but also the trajectory of its change in space [52].

If the change process has *P* classifications, the study object may have *P* states at the same time point; if the study object intercepts *q* time phases, the study object has *P_q_* spatio-temporal change trajectory [44]. The spatio-temporal change trajectory is expressed by the following raster calculation formula:

(6) $$C=P_{1}\times 10^{q-1}+P_{2}\times 10^{q-2}+\cdots +P_{i}\times 10^{q-i}+\cdots +P_{q}$$

where *C* denotes the spatio-temporal trajectory code corresponding to the study object on the whole timeseries, *q* denotes the number of time phases within the whole timeseries (*q* > 1), *P_i_* denotes the raster data of the study object corresponding to the *i*-time phase. The following is the precise procedure: (1) create a map from the index vector distribution map (the indicators in this study are the Weibo emotion score, hazy perception index, and psychological tolerance index); (2) reclassify the raster data according to the index’s specific value (usually separated into 5 groups with 1–5 values assigned to each); and (3) use the ArcGIS raster calculation tool and the aforementioned algorithm to calculate the temporal and spatial trajectory change code of raster elements.

## 3. Results and Discussion

### 3.1. Sentiment Analysis

**Figure 3:**
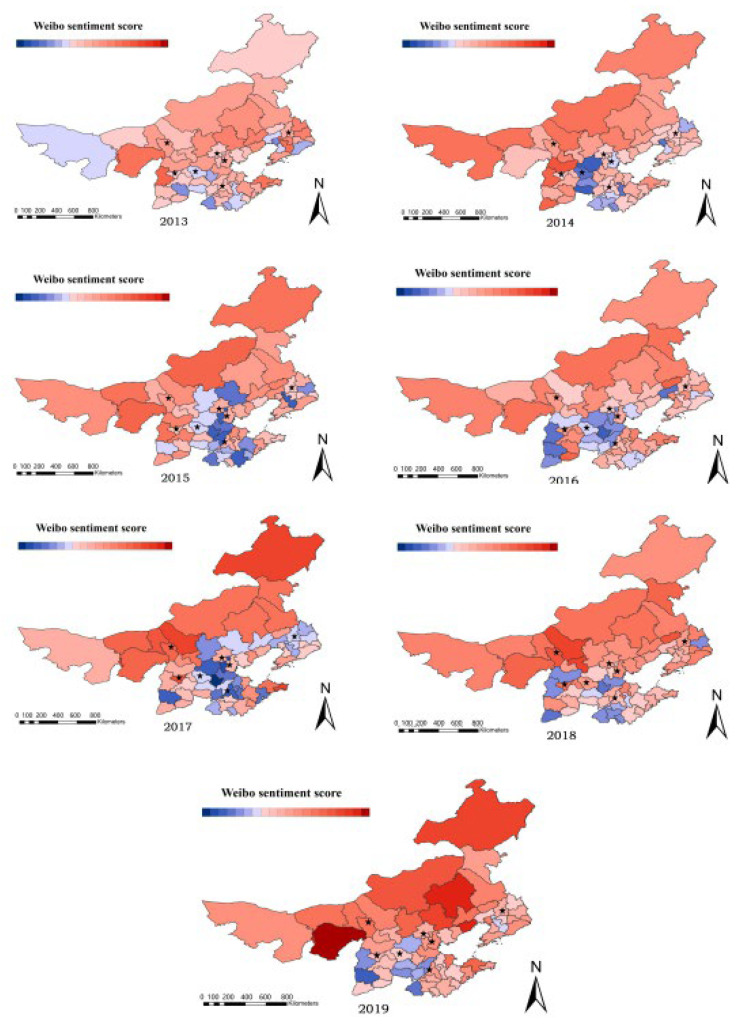
The temporal and spatial dispersion of the sentiment score (red (+); blue (−)). (The stars indicate the capitals of seven provinces).

**Figure 4:**
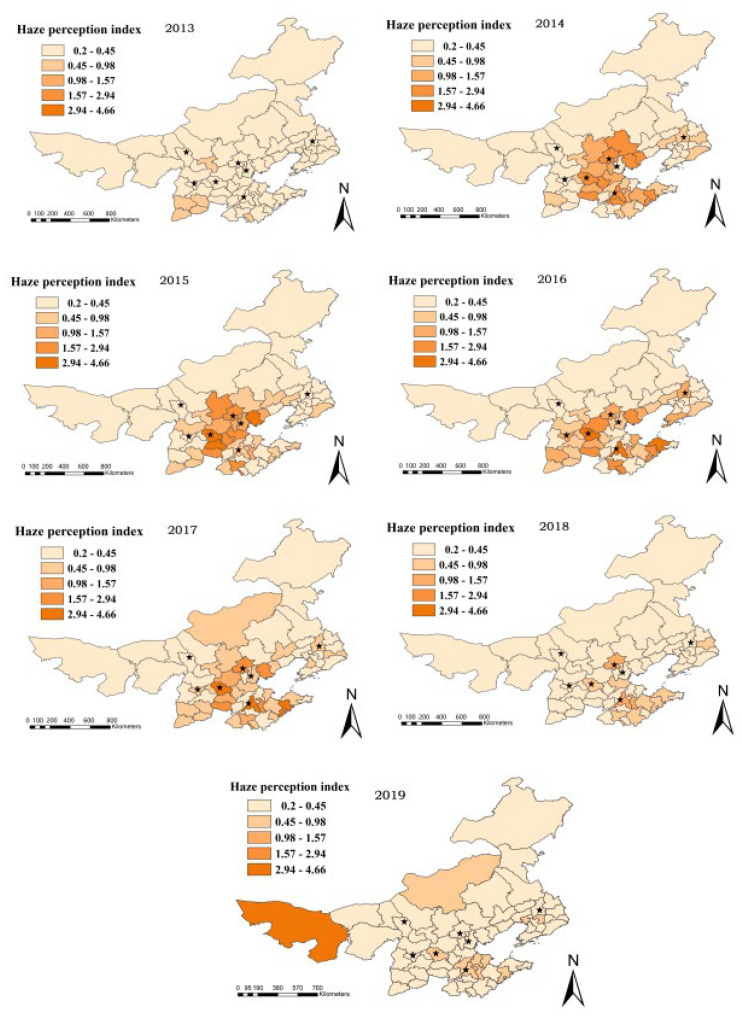
Perception of haze on a temporal and spatial scale. (The stars indicate the capitals of seven provinces).

**Figure 5:**
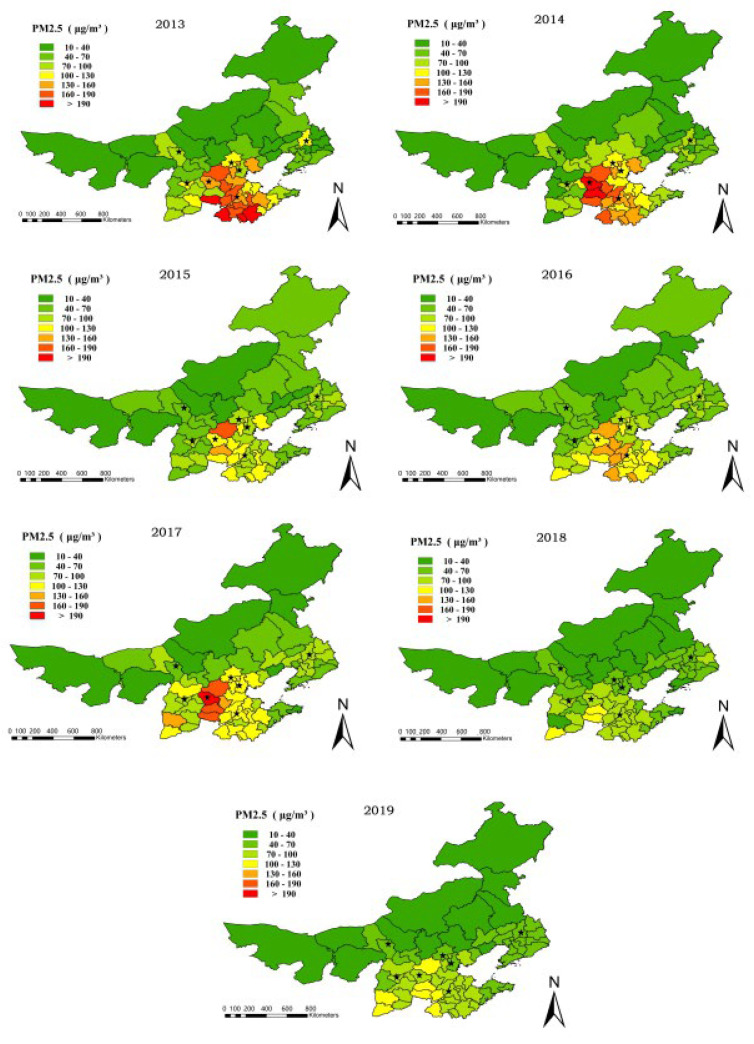
PM2.5 concentrations have a temporal and spatial distribution. (The stars indicate the capitals of seven provinces).

We obtained the result of the emotion value by SnowNLP sentiment analysis, which is presented in Table 4. The Weibo data utilized to construct the emotion score have a very low temporal resolution. Since PM2, the sentiment score of seven provinces and cities in the winter from 2013 to 2019 was calculated using the same temporal resolution. We estimated the average sentiment score for the entire winter using five concentrations spread on a seasonal scale. ArcGIS was used to create a regional and temporal distribution map of emotion based on the emotional calculation findings of seven provinces and cities, as shown in Figure 3. At the same time, the haze perception index was created, which has the same temporal resolution as the sentiment score, and the temporal resolution is season, and the spatial and temporal distribution of PM2.5 concentration and haze perception index was created, which has the same temporal resolution as the sentiment score, and the temporal resolution is season, as shown in Figure 4 and Figure 5. Seven province capitals are represented by the stars.

Figure 3 shows that red denotes a good sentiment score, blue denotes a negative sentiment score, and the color depth denotes the degree of emotion. The research area included 66 cities. Red signifies a positive sentiment score, blue denotes a negative sentiment score, and the color depth denotes the degree of emotion, as seen in Figure 2. Dark red, for example, elicits a more pleasurable response than bright red. Based on the precise circumstances of emotion score, this study divides (0.36–0.76) into 20 cells (the minimum value is 0.36 and the maximum value is 0.76). The number of negative cities increased, then decreased, then increased, then decreased from 2013 to 2019, with a trend of first increasing, then dropping, then increasing, and then declining. Overall, the number of cities with negative emotions is dropping, which is the inverse of the sentiment score trend. The more cities with negative emotions, the more unpleasant the total sentiment. In a horizontal comparison, Inner Mongolia has the happiest feelings, but the other regions have good and negative emotions that are staggered.

Figure 4 demonstrates that, on average, people’s perceptions of haze intensity grew stronger from 2013 to 2015 and then steadily diminished from 2016. In a horizontal comparison, the degree of haze perception changes as haze pollution and people’s comprehension of the phenomena varies. People’s perceptions of haze were a little behind in the early stages of haze pollution. Following the formal announcement, haze became associated with catastrophic weather, and the degree of hazy perception increased dramatically. As more individuals acknowledge the occurrence of haze, the amount of discussion about it reduces, resulting in a weakening of haze perception.

Figure 5 shows that from 2013 to 2019, PM2.5 concentrations were 90, 88, 75, 80, 86, 60, and 67 (μg/m^3^), and air quality first increased, then decreased, then increased, and then decreased, which was counter to the change rule of microblog emotion, indicating that microblog emotion was negatively correlated with PM2.5. The pollution degree in the Beijing-Tianjin-Hebei region and several cities in Shandong province is generally higher than that of other regions according to a horizontal comparison of spatial differences. The worst-affected province is Hebei, whereas Inner Mongolia has the finest air quality.

### 3.2. Local Keyword Analysis

**Figure 6:**
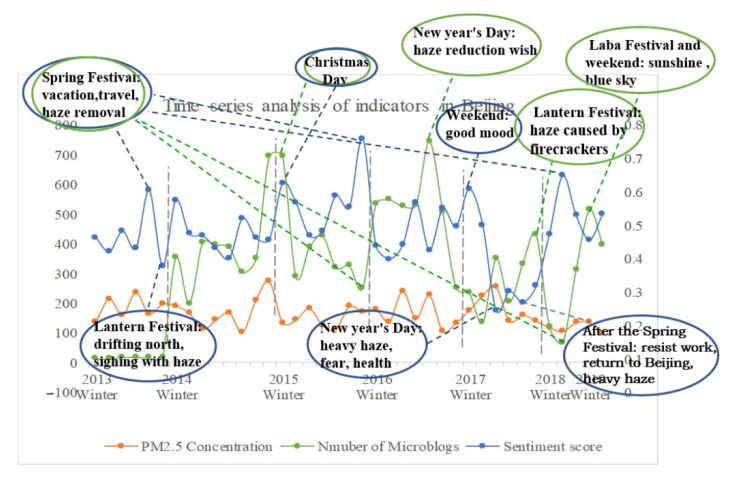
Indicator timeseries study in Beijing.

PM2.5, microblog quantity, and microblog sentiment score were all subjected to timeseries analysis in this study. When the prefecture-level city was chosen as the spatial scale, the data volume was vast and difficult to gather, resulting in a large number of missing values, which made the experimental analysis challenging. As a result, the province was selected as the geographical scale for conducting a timeseries analysis of associated indicators in each province in the research region. Referring to the literature [53], text keywords were extracted using the TF-IDF keyword extraction algorithm, and the TF (term frequency) value, IDF (inverse document frequency), and the product of the TF-IDF value for preprocessed Weibo words were calculated using Python. The higher the value, the more likely it is that the terms will be used as keywords. The following conclusions were drawn from keyword analysis of the local peak of the curve (Figure 6): Each indicator’s extreme values come around holidays or major time nodes. As an example, consider Beijing: People are often in a good mood during the holidays and express their concerns about haze in various ways, such as the desire to minimize haze and the satisfaction of seeing the haze go. However, when people return to Beijing after their vacations, especially after the Spring Festival, some people will be depressed. For example, after the Spring Festival, most people dread work, groan about the haze in Beijing, and fear the health problems produced by the smog as opposed to the holidays at home. Holidays, it can be observed, are the differentiating node in people’s perceptions of haze. People are more likely to express favorable views during the holidays, but their feelings are unpleasant afterward, and they express negative views.

### 3.3. Psychological Tolerance

Haze is a prevalent form of bad weather in China, especially in the autumn and winter. Several studies have found that haze pollution harms human mental health by interfering with hormone secretion. Mental health is crucial; yet, it is easy to overlook it. The majority of present research focuses on the causes and mechanisms of haze pollution as well as the impact of haze on production and life, with only a few studies examining the influence of haze on people’s mental health. The psychological tolerance index is offered in this study to quantify people’s psychological tolerance. To examine the provincial spatial difference, the weight of 18 indicators was measured at the province level using the AHP-entropy weight approach. Relevant indicators were chosen at the prefecture-level city level to construct the psychological tolerance index model, which serves as a theoretical foundation for the development of a psychological early warning mechanism.

#### 3.3.1. Provincial Scale

(1) Evaluation Index System

Three system layers, namely ecological environment, social economy, and social media, were finally selected to establish an evaluation system and construct a hierarchical structure model based on the principles of science, typicality, operability, and systematicity, reviewing relevant literature, and considering all factors. The psychological tolerance index is the first layer; the second layer is the system layer, which includes three first-level indicators, namely ecological environment, social economy, and social media; and the third layer is the indicator layer, which includes a total of 18 second-level indicators under each system layer, as shown in Table 5.

The ecological data were obtained from the Climate Data Center, the social and economic data were obtained from the city statistics yearbook, and the social media data were statistically analyzed based on the foregoing findings. Because the indices used in the study pertain to different dimensional levels, the range standardization method was chosen to compensate for this.

(2) Matrix of Index Judgment

This study asked 10 experts to score and develop four judgment matrices, including one first-level indicator judgment matrix as given in Table 6 and three second-level indicator judgment matrices as shown in Table 7, Table 8 and Table 9, to ensure the rationality of index scoring.

(3) Calculation of Feature Vectors and a Consistency Test

The CR and CI values of the four judgment matrices are less than 0.1, indicating that the judgment matrices are consistent, and weights are produced for these judgment matrices by calculating the feature vector. After calculating the target layer’s weights, the criteria layer’s intra-group weights can be calculated, and the product of the two is the criterion layer’s final absolute weight value.

(4) Weight of the Combination

Calculate the index entropy weight and comprehensively calculate it with the weight of analytic hierarchy process, and the weight of each index factor in the evaluation system was obtained, and the calculation results are shown in Table 10.

**Figure 7:**
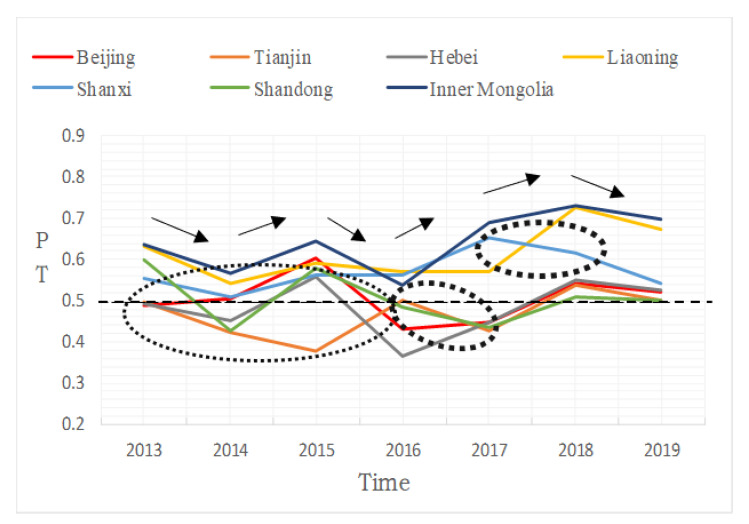
Timeseries of tolerance index.

By multiplying the comprehensive weight value $\omega_{i}$ with the standard normalized index value *x_i_*, we can obtain the psychological tolerance index in the haze situation: $z_{i}=\overset{m}{\underset{i=1}{\sum }}\omega_{i}x_{i}$, *i* = 1, 2, 3,…, *m*. The results of the calculation are shown in Table 11, and the timeseries analysis of the obtained indexes is shown in Figure 7.

Figure 7 shows that: (1) from 2013 to 2019, the psychological tolerance of provinces and cities in the study region decreased-increased-decreased-increased-increased-increased-decreased (As shown by the arrow trend in the Figure 7); (2) abnormal psychological tolerance developed in various periods; for example, in 2015, Tianjin’s psychological tolerance reached an all-time low, and Shandong and Shanxi provinces also displayed a unique changing rule, indicating that their tolerance differed from that of other regions. Shandong province’s psychological tolerance decreased in 2016, and Shanxi province’s psychological tolerance decreased in 2017 (As shown by the dotted ellipse in the figure). Other provinces and cities’ psychological tolerance grew within the same time period; (3) the degree of tolerance can be divided into two groups using 0.5 as the threshold. Shanxi Province, Liaoning Province, and Inner Mongolia are among the regions with a high level of tolerance, whereas the other four provinces have a low level of tolerance.

#### 3.3.2. Prefecture Level City Scale

**Figure 8:**
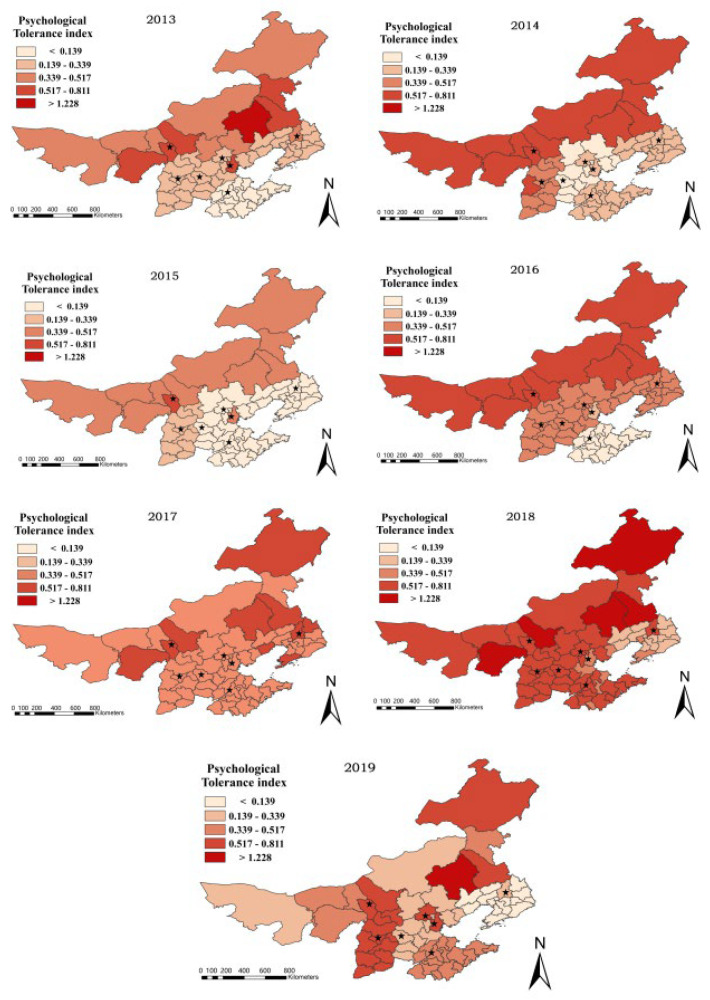
Psychological tolerance’s temporal and spatial distribution in prefecture-level cities. (The stars indicate the capitals of seven provinces).

The haze psychological tolerance index was calculated using the Formula (5), and a comprehensive evaluation grade classification map was created using ArcGIS. The evaluation results are displayed in Figure 8.

### 3.4. Analysis of Spatiotemporal Trajectories

**Figure 9:**
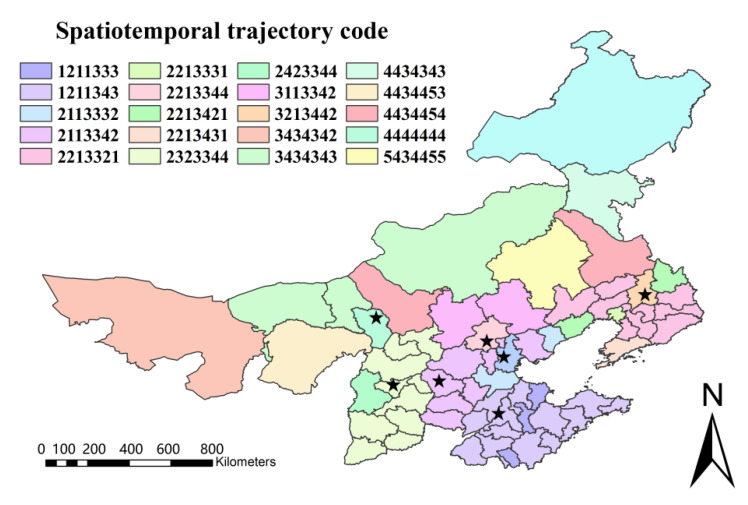
Temporal and spatial changes of prefecture level city scale psychological tolerance in winter 2013–2019. (The stars indicate the capitals of seven provinces).

**Figure 10:**
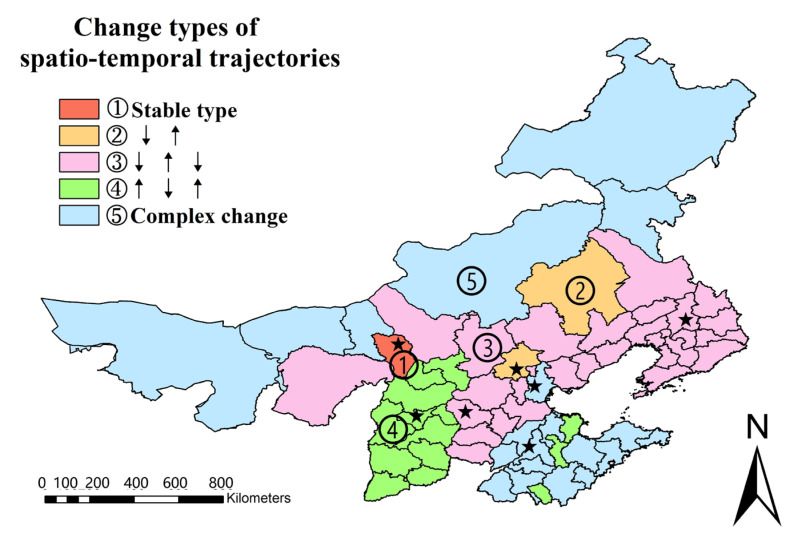
Psychological tolerance index types with spatial and temporal variation. (The stars indicate the capitals of seven provinces).

Vector rastering, reclassification, raster calculation, and other operations are performed on the spatiotemporal analysis results of psychological tolerance using the spatio-temporal trajectory method (Formula (6)) to obtain the spatial and temporal trajectory changes of prefecture-level city-scale psychological tolerance, as shown in Figure 9 and Figure 10.

In Figure 9, code 1 denotes a low tolerance, code 2 a lower tolerance, code 3 a moderate tolerance, code 4 a higher tolerance, and code 5 a high tolerance. The findings of the spatio-temporal trajectory analysis reveal that the spatio-temporal trajectory of psychological tolerance at the prefecture-level city scale is complicated, with 20 different types of spatio-temporal trajectory alterations.

## 4. Discussion

Figure 8 demonstrates that, when all prefecture-level cities in the study area’s psychological tolerance values are added together, the psychological tolerance indexes under the prefecture-level city scale from 2013 to 2019 are 0.273607, 0.339138, 0.167824, 0.385077, 0.461151, 0.552613, and 0.382478, indicating the trend of increasing, decreasing, increasing, increasing, increasing, increasing, and decreasing. In contrast, the horizontal, which includes the number of microblogs, emotions, and a measure of psychological tolerance at the prefecture-level city scale, reveals that people’s psychological tolerance does not gradually improve as haze pollution improves but rather fluctuates based on a variety of influencing factors.

The spatio-temporal trajectory changes of psychological tolerance at the scale of prefecture-level cities can be categorized into five types, as shown in Figure 9 and Figure 10: no change type, increase–decrease type, decrease–increase–decrease type, increase–decrease–increase type, and complex change type: (1) The no change type denotes no change in psychological tolerance, and cities that fall under this category include Hohhot; (2) the decline–rise type shows a decrease in psychological tolerance followed by an increase, and cities in this category include Beijing, Chifeng, and Inner Mongolia; (3) the decline–rise–decline type denotes a decrease in psychological tolerance, followed by an increase and lastly a decrease, and cities that fall under this category include Qinhuangdao, Cangzhou, Shijiazhuang, Tangshan, Handan, Xingtai, Baoding, Langfang, Hengshui, Zhangjiakou, Chengde, Hebei Province, Anshan, Fushun, Benxi, Dandong, Jinzhou, Yingkou, Fuxin, Liaoyang, Chaoyang, Panjin, Tieling, Huludao, Dalian, Shenyang City, Inner Mongolia, Erdos, Tongliao, and Ulanqab; (4) the rise–decline–rise type denotes that psychological tolerance increases first, then decreases, and then increases again, and those cities are Taiyuan, Datong, Yangquan, Changzhi, Jincheng, Shuozhou, Jinzhong, Yuncheng, Xinzhou, Linfen and Luliang in Shanxi Province, Zibo, Zaozhuang, and Dongying in Shandong Province; (5) the amount of psychological tolerance evolves in a complex manner, and cities of this sort are characterized by complex transformation: Tianjin, Jinan, Qingdao, Yantai, Weifang, Jining, Tai’an, Weihai, Rizhao, Linyi, Dezhou, Liaocheng, Binzhou, and Heze in Shandong Province and Alxa League, Baotou, Wuhai, Bayannur, Xilin Gol League, Hulunbuir, and Xing’an League in Inner Mongolia were characterized by such complexity.

## 5. Conclusions

The SnowNLP model and AHP-entropy weight method were used to investigate the impact of haze events on urban crowd activities in seven provinces and cities in north China, east China, and northeast China. The virtual space was used as the research perspective, and the SnowNLP model and AHP-entropy weight method were used to investigate the influence of haze events on urban crowd activities.

In conclusion, we found that the spatial-temporal distribution of microblog emotion, hazy perception, and PM2 are all related to each other. Five indicators were mapped, and it was discovered that each indication has its own unique temporal change patterns while also having a correlation with one another. In general, the more significant the haze pollution, the more negative people’s emotions are, and the more heated the haze discussion is. The relaxation and joyous environment of the holidays, on the other hand, causes a reversal of feeling in haze situations, and the issues of concern on haze events become more cheerful.

The psychological tolerance of haze pollution does not increase gradually as haze pollution improves but rather shows a variety of complex changes as haze pollution progresses; the results of spatio-temporal trajectory analysis show that there are five types of changes in psychological tolerance at the municipal scale, namely no change, decrease–increase, increase–decrease–increase, decrease–increase, and complex change.

This study focused on existing indicators for the quantitative assessment of psychological tolerance, and through the collection of existing indicators, an assessment index system was built, and the quantitative modeling of psychological tolerance was finally completed, which is useful for understanding the population’s psychological tolerance level during the development of haze pollution. However, because we only examined historical data and did not investigate future development, a significant amount of work remains to model and train the development of psychological tolerance in a hazy condition in order to anticipate future development.

## Figures and Tables

**Table 1:** Text preprocessing on a microblog.

Original Microblog	Cleaned Microblog	Remove Stop Words
What businesses have been closed or banned as a result of the recent blue sky cloudy weather?	The hazy weather has been substantially reduced in return for the current blue sky, and businesses have been shut down or outlawed.	Many businesses have closed as a result of the recent hazy weather, and the current blue sky has been outlawed.
Toxins and harmful substances are eliminated from the body by enzymes that serve no other purpose but to keep the body healthy; consequently, health comes first!	During highly hazy days, enzymes are required to remove toxins and hazardous chemicals from the body; therefore, health comes first.	On days when there is a lot of haze, enzymes remove toxins and poisonous substances from the body.
Haze is made up of a variety of substances that will most likely thicken in the skin! (Puhuangyu Community Health Center)	Haze has several substances that will likely build up in the skin.	The substances that cause haze block skin.

**Table 2:** Criteria for scoring in the judgment matrix.

Comparative Score	Relative Importance	Explanation
1	Equally crucial	Indicates that both variables are equally important
3	Significantly important	One element has a tiny advantage over another
5	Important	One component takes precedence over another
7	Extremely significant	One aspect is far more significant than the other
9	Extremely crucial	One aspect is far more crucial than the others
2, 4, 6, 8	Adjacency has a middle value.	When a compromise between the two components is required, these values are employed

**Table 3:** The RI is the average random consistency index.

Matrix Order	1	2	3	4	5	6	7	8	9
*RI* value	0	0	0.58	0.90	1.12	1.24	1.32	1.41	1.45

**Table 4:** Example of calculation result of emotion value of microblog data.

Cleaned Microblog	Release Time	Sentiment Score	Sentiment Tendency
There are no foggy days in life as long as the heart is clear. New Year’s greetings for 2017!	1 January 2017, 09:44	0.710162602	Positive
The haze is such that still have the heart to fire the gun; I have nothing to say.	1 February 2017, 08:01	0.32582773	Negative
On a polluted day, high school students acted as nature’s vacuum cleaners, while teachers stayed with their haze masks.	4 May 2017, 08:57	0.460606061	Negative
This winter haze days really too much less awesome.	18 December 2017, 11:56	0.809009009	Positive

**Table 5:** Evaluation index system.

Target Layer	Rule Layer	Index Layer
Psychological tolerance (A)	Ecological environment (B1)	Air quality (μg/m^3^, C1), temperature (°C, C2), relative humidity (%, C3), wind speed (m/s, C4), pollution days (d, C5)
	Social economy (B2)	Population density (person/km^2^, C6), per capita GDP (CNY, C7), proportion of secondary industry (%, C8), proportion of built-up area (%, C9), road area (10,000 m^2^, C10), per capita green space area (m^2^, C11), education level (C12), gender ratio (female = 100, C13)
	Social media (B3)	Number of microblogs (C14), correlation coefficient of number (C15), sentiment score (C16), correlation coefficient of sentiment (C17), topic index (C18)

**Table 6:** Primary index judgment matrix.

Primary Index	Ecological Environment	Social Economy	Social Media
Ecological environment	1	4	0.33333
Social economy	0.25	1	0.14286
Social media	3	7	1

**Table 7:** Secondary index judgment matrix 1.

Secondary Index	Temperature	Air Quality	Relative Humidity	Wind Speed	Pollution Days
Temperature	1	5.95733	5.37924	6	1
Air quality	0.16786	1	0.36	2.35832	0.34832
Relative humidity	0.18591	2.77778	1	2.63424	0.13234
Wind speed	0.166667	0.42399	0.37962	1	0.16982
Pollution days	1	2.87092	7.55629	5.88859	1

**Table 8:** Secondary index judgment matrix 2.

Secondary Index	Population Density	Per Capita GDP	Proportion of Secondary Industry	Proportion of Built-Up Area	Road Area	Per Capita Green Space Area	Education Level	Gender Ratio
Population density	1	0.68472	0.15402	0.65873	0.52938	0.62348	0.31245	0.53981
Per capita GDP	1.46045	1	0.28564	2.43921	2.32913	0.31569	0.29837	2.4
Proportion of secondary industry	6.49284	3.50091	1	2.5	1	2.47921	2.68931	2.78931
Proportion of built-up area	1.51807	0.40997	0.4	1	0.5	0.33333	0.57532	0.5
Road area	1.88901	0.42934	1	2	1	0.7	0.65	2.63922
Per capita green space area	1.60391	3.16766	0.40335	3	1.42857	1	2.42324	2.58482
Education level	3.20051	3.35154	0.37184	1.73816	1.53846	0.41267	1	2.35382
Gender ratio	1.8525	0.41667	0.35851	2	0.37879	0.38687	0.42484	1

**Table 9:** Secondary index judgment matrix 3.

Secondary Index	Number of Microblogs	Correlation Coefficient of Number	Sentiment Score	Correlation Coefficient of Sentiment	Topic Index
Number of microblogs	1	0.31582	0.166667	0.45225	0.12424
Correlation coefficient of number	3.16636	1	0.65392	1.1	0.16667
Sentiment score	6	1.52924	1	1.27	0.72737
Correlation coefficient of sentiment	2.21116	0.90909	0.7874	1	1.1
Topic index	8.04894	6	1.37482	0.90909	1

**Table 10:** Calculation results of the index weight.

Target Layer	System Layer	Index Layer	AHP	Entropy Weight Method	Combination Weight
Psychological tolerance (PT)	Ecological Environment (B1)	Air quality (C1)	0.103307	0.01794	0.070799
		Temperature (C2)	0.021266	0.087627	0.046536
		Relative humidity (C3)	0.027740	0.059598	0.039871
		Wind speed (C4)	0.012561	0.07623	0.036806
		Pollution days (C5)	0.098562	0.054243	0.081686
	Social economy (B2)	Population density (C6)	0.029542	0.114473	0.061884
		Per capita GDP (C7)	0.004224	0.033572	0.015400
		The proportion of secondary industry (C8)	0.008478	0.070581	0.032127
		The proportion of built-up area (C9)	0.020486	0.064344	0.037187
		Road area (C10)	0.004855	0.068431	0.029065
		Per capita green space area (C11)	0.009563	0.050828	0.025277
		Education level (C12)	0.014204	0.055002	0.029740
		Gender ratio (C13)	0.011559	0.024712	0.016568
	Social media (B3)	Number of microblogs (C14)	0.033488	0.022391	0.029262
		The correlation coefficient of number (C15)	0.090235	0.03637	0.069723
		Sentiment score (C16)	0.159775	0.034084	0.111912
		The correlation coefficient of sentiment (C17)	0.124904	0.096453	0.114070
		Topic index (C18)	0.249201	0.033121	0.166918

**Table 11:** Comprehensive evaluation results of psychological tolerance.

	2013	2014	2015	2016	2017	2018	2019
Beijing	0.48971	0.506841	0.602236	0.432072	0.447645	0.541737	0.521512
Tianjin	0.495376	0.422032	0.378095	0.501404	0.426962	0.53912	0.498869
Hebei	0.491715	0.453081	0.557138	0.363952	0.446847	0.548772	0.527312
Liaoning	0.632011	0.539867	0.591269	0.569133	0.569305	0.72548	0.674516
Shanxi	0.553621	0.510981	0.564137	0.561731	0.650882	0.613825	0.542213
Shandong	0.598622	0.426567	0.576922	0.48618	0.434261	0.50842	0.502466
Inner Mongolia	0.637273	0.566498	0.644165	0.539332	0.689162	0.731031	0.697857

## Data Availability

The air quality data we used are from the air quality index website and are available online at (https://aqicn.org/map/china/cn/) (accessed on 16 April 2020). The air microblog data we used are from the Sina Weibo platform and available online at (https://s.weibo.com/weiboq=%E9%9B%BE%E9%9C%BE&Refer=index) (accessed on 18 July 2020).
